# The Treatment of Fractures

**Published:** 1905-12-16

**Authors:** 


### The Treatment of Fractures.

It may be taken as an axiom that fractures are among the most troublesome cases which the medical practitioner is called upon to treat. Many a fair professional reputation has crumbled away beneath the opprobrium called up by a single badly united broken bone. For it is an unfortunate circumstance that the average citizen commonly holds the opinion that a limb which has sustained a fracture ought to be completely restored in the course of a few weeks or months to its former state of efficiency and proper appearance. Anything short of a perfect result is liable to be attributed to incapacity or negligence on the part of the medical attendant. In consequence of this idea the "badly set" limb is for ever being displayed as a testimony to some poor doctor's carelessness or lack of skill. The advent of the *x*-ray photograph has accentuated rather than diminished the surgeon's difficulties. For often what appears to ordinary inspection as a complete restoration of normal form to a limb which has been the seat of a fracture, shows, when examined by the *x*-rays, that the fragments of bone are far from being in exact apposition; and, unless he has been warned beforehand, this apparent deformity is likely to fill the patient with consternation and dismay. Doubtless considerations such as these have been mainly responsible for the vigour and enthusiasm with which certain surgeons are now advocating the open method of treating fractured bones. To cut down upon the fragments and fix them accurately in position appeals with much force to the anatomical sense, and is certainly a very reasonable procedure in cases of compound fracture. But the proposition to adopt this measure as a routine practice in the treatment of so-called simple fractures is not likely to commend itself to the judgment of either the laity or the more soberminded members of the profession. For the operation is not devoid of danger or free from drawbacks, even in the hands of the most skilful and painstaking operators. Certainly undue confidence in the operation is to be deprecated, and no patient should be advised to have a fracture wired without being made fully aware of the risks that are inseparable from the operation. The surgeon who aspires to be a master of his art must be, like Sydenham, a man of many doubts. Such a one will not embark his patient on a perilous venture without that infinite carefulness and forethought which are inseparable from good surgery.

We do not mean that the wiring of simple fractures is never justifiable. But we do strongly oppose any attempt to recommend this treatment as a routine measure, apart from the fact that routine surgery is bad surgery. Cases differ widely, and each must be considered on the ground of its own peculiarities. Doubtless there is a small proportion of simple fractures for which the best treatment is the open operation, provided always that the surgeon is competent to reduce the chances of septic infection to a minimum. For the remainder, the safer methods of the past must suffice.

Many of the drawbacks attaching to the usual treatment of fractures can be avoided. Careful examination of the injured part, with the help of the *x*-rays whenever possible, sharing the responsibility by consultation with a colleague, careful treatment, with the use of a general anæsthetic when "setting the fracture," and a very guarded prognosis in all cases. These precautions will considerably diminish the many anxieties arising from the treatment of an injury which our forefathers most injudiciously termed a simple fracture.

